# Australian general practitioners’ knowledge, attitudes and prescribing intentions for e-cigarettes as a smoking cessation aid: a nationwide baseline and 12-month follow up survey

**DOI:** 10.1186/s12954-025-01175-2

**Published:** 2025-03-18

**Authors:** Melis Selamoglu, Sowmya Malamardi, Bircan Erbas, Hester Wilson, Jamie Brown, Chris Barton

**Affiliations:** 1 Department of General Practice, School of Public Health and Preventive Medicine, Monash University, Melbourne, Australia; 2 Department of Public Health, School of Psychology and Public Health, La Trobe University, Melbourne, Australia; 3 Population and Community Health, South Eastern Sydney Local Health District, Sydney, Australia; 4 School of Population Health, University of New South Wales, Sydney, Australia; 5 Centre for Primary Health Care and Equity, University of New South Wales, Sydney, Australia; 6 Department of Behavioural Science and Health, University College London, London, UK

**Keywords:** E-cigarette, Smoking cessation, Primary care, General practice, Harm reduction, Public health

## Abstract

**Background:**

The Australian Government introduced a major policy change tightening regulations regarding the access to nicotine containing e-cigarettes in October 2021. We assessed general practitioners’ (GPs) knowledge, attitudes, beliefs and intentions to prescribe nicotine containing e-cigarettes for smoking cessation. We compared baseline data near the time of policy change with data collected from these GPs 12-months later.

**Methods:**

GPs were invited to complete a repeated cross-sectional survey based on the Theory of Planned Behaviour between December 2021 and March 2022 (T1) and again, between January and April 2023 (T2). Survey questions assessed knowledge, attitudes, beliefs and intention to prescribe e-cigarettes for smoking cessation.

**Results:**

A total of 264 GPs completed the baseline (T1) survey and 94 provided responses at follow-up (T2). Over half of responders were female (T1 *n* = 170, 64.4%, T2 *n* = 57, 60.6%) and roughly one third were aged between 30 and 39 years (T1 *n* = 80, 37.2%, T2 *n* = 28, 29.8%). Participants who agreed e-cigarettes were suitable smoking cessation aids were more willing to recommend e-cigarettes to patients at T1 and T2 (T1 *n* = 29, 87.9%, vs. T2 *n* = 20, 100%). Knowledge about e-cigarettes was limited and did not change between T1 and T2. Participants who had greater confidence in their ability to talk to and answer patient questions about e-cigarettes were more likely to recommend them for smoking cessation at both T1 and T2 (T1 *n* = 24, 70.6% vs. T2 *n* = 17, 85.0%).

**Conclusion:**

Since tightening the regulation of nicotine containing e-cigarettes, there has been little change in Australian GPs’ perceptions of e-cigarettes as smoking cessation aids. Australian GPs are poorly educated about vaping and knowledge about e-cigarettes remained limited, however, GPs at follow-up were more confident in their ability to discuss e-cigarettes with their patients. The findings from this survey may help guide policy and develop strategies to support the implementation of smoking cessation guidelines that incorporate the use of e-cigarettes as smoking cessation aids in Australia.

**Supplementary Information:**

The online version contains supplementary material available at 10.1186/s12954-025-01175-2.

## Background

Tobacco smoking is the leading cause of preventable death worldwide and a major risk factor for non-communicable diseases such as cancer, cardiovascular and respiratory diseases [1]. More than 8 million deaths per year are a result from smoking-related illnesses, with 7 million deaths occurring from a direct link with tobacco use and 1.3 million deaths caused by second-hand smoke exposure [1, 2]. Recent data from 2020 shows that, nearly 1.2 billion people smoke tobacco, with a global smoking prevalence of 32.6% among adults [3]. The use of electronic cigarettes (e-cigarettes) globally continues to rise significantly each year, and they may potentially have a role with reducing the health risks associated with tobacco smoking and aiding in smoking cessation [4]. Data from 2018 estimated that 58.1 million people were vaping globally, increasing to 68 million people who vaped in 2020 to 81.9 million in 2021 [5, 6].

E-cigarettes are devices that are powered by a battery and heat liquids, usually containing nicotine, to produce a vapour which is inhaled by the person who vapes and is more commonly known as ‘vaping’ [7]. The Australian Government introduced a major policy change regarding the use of nicotine containing e-cigarettes in October 2021 [8]. Prior to the law changing, individuals were able to purchase nicotine vaping products from overseas websites with a nicotine prescription [8]. The policy changes in Australia were introduced to limit the rapid increase in the number of young adults who used e-cigarettes in the community, and in particular, access to nicotine e-liquids [8]. In 2022–2023, the prevalence of young adults aged between 18 and 24 years who currently used e-cigarettes increased substantially from 5.3% in 2019 to 21% [9]. Similarly, among individuals aged between 14-17 years, this rose from 2.5% (2019) to 7% (2022–2023), with curiosity being one of the main reasons for using e-cigarettes among both these age groups [9]. Following this regulatory change, nicotine containing e-liquids could only be legally obtained with a prescription from a registered general practitioner (GP) and recently, a nurse practitioner from January 1, 2024 [10].

For some people who smoke, GPs are the first point of contact to seek information, advice, treatment and support to help them quit smoking [11]. Smoking cessation guidelines by the Royal Australian College of General Practitioners (RACGP) recommend using the three-step brief intervention model (Ask, Advise and Help) to encourage people who smoke to quit smoking [12]. These guidelines recommend the initial offer of first line smoking cessation therapy such as nicotine replacement therapies (NRTs), varenicline and bupropion as well as behavioural support, and only recommend e-cigarettes with behavioural support as second-line therapy [12].

In Australia, e-cigarettes have not been approved by the Therapeutic Goods Administration (TGA) as devices for smoking cessation however, there are four pathways that GPs can use to prescribe nicotine containing e-cigarettes as therapy in combination with behavioural support [10, 12].

From July 1, 2024 pharmacies will have the authority to distribute and supply e-cigarettes to customers [13]. From October 2024, individuals aged 18 years and older are able to access nicotine containing e-cigarettes (up to and including 20 mg/mL nicotine) from pharmacists without a prescription however, those aged below 18 or adults requiring greater than 20 mg/mL of nicotine will need a prescription from a GP or nurse practitioner [13].

Recent qualitative studies with Australian GPs [14] and health professionals [15], and a systematic review of international literature [16] found that, GPs had mixed views about e-cigarettes and were uncertain about their intentions to prescribe e-cigarettes as an alternative to other, more established smoking cessation methods. They lacked the knowledge and confidence to have discussions with patients about e-cigarettes and held concerns about their safety and efficacy as smoking cessation aids [14–16].

Similarly, studies conducted in other countries, found that physicians, GPs and nurse practitioners lacked knowledge about e-cigarettes and reported high levels of uncertainty in recommending e-cigarettes as an aid to smoking cessation, due to their perceptions about limited evidence on their safety and efficacy of e-cigarettes as alternative smoking cessation methods [17–22]. Even in the UK, where guidelines such as the National Institute for Health and Care Excellence promote their use as a smoking cessation aid [23], only 4% of people who smoke that visited their GP reported being recommended an e-cigarette for smoking cessation [24].

To the best of our knowledge this is the first study in Australia to explore Australian GPs’ knowledge, attitudes, beliefs and intentions to prescribe nicotine for use with e-cigarettes as smoking cessation aids and compared baseline data (T1) collected at the time of regulatory change in 2021 with data collected 12-months later (T2).

## Methods

### Participants and recruitment

A nationwide repeated cross-sectional survey of GPs in Australia was conducted between December 2021 and March 2022 (T1) and again between January and April 2023 (T2). Australian GPs from all states and territories were invited to complete an online electronic self-administered survey created using Qualtrics™. The questions were adapted from published literature [20, 25–31] and face-validity was considered by six GPs selected from within the Department of General Practice at Monash University as part of the pilot testing of the survey. The survey was distributed through practice-based research networks, primary health networks and social media platforms.

A participant information sheet was presented to participants upon clicking the survey link. Participants could then proceed to commence and complete the survey questions. Participation was voluntary and consent was obtained once participants began to proceed with the survey. Participants were able to exit the survey at any point if they no longer wished to continue but their data was unable to be removed due to the anonymous nature of data collection.

Participants were offered to enter a prize draw at the end of the survey to win one of three $500 gift vouchers and were taken into a separate survey to provide their contact details. The answers provided by participants in the survey and the prize draw were not linked to maintain participant anonymity.

This study was approved by the Monash University Human Research Ethics Committee (ID:28552).

### Survey and measures

Survey questions were grouped based on the Theory of Planned Behaviour (TPB) [32] to explore and understand prescribing intentions and behaviours of GPs. The TPB is based on the premise that individuals make logical, reasoned decisions to engage in specific behaviours by evaluating the information available to them. The TPB has three domains, (i) attitudes (ii) subjective norms and the influence of social pressure and (iii) perceived behavioural control. All three can affect the intention to undertake a behaviour and strength of intention can impact on whether an individual performs the behaviour [32].

The survey contained 22 items. Nine collected sociodemographic information from participants. These included age, gender, postcode of practice, the type of practice (solo, group, corporate, Aboriginal health services), years in clinical practice, the country of medical training, types of qualifications held and if participants smoked or used e-cigarettes. A series of questions assessed the beliefs of GPs around the safety and efficacy of e-cigarettes as a smoking cessation aid. These were presented on a five-point Likert scale with options ranging from “strongly disagree” to “strongly agree” and were adapted from previous surveys [20, 26, 29–31]. We tested GPs’ knowledge about e-cigarettes and included six questions adopted from Moysidou et al. [27] that asked GPs, “In your opinion which of the following are correct concerning e-cigarettes in Australia”, with a selection of options ranging from “Yes, Unsure, No”. Each correct answer was scored as plus one with a maximum total of six. The correct answers to the questions can be found in the supplementary file of S1 and S2, Q10. Scores were categorised as low if the knowledge scores were less than or equal to the average median scores and high if above. Another question was modified from Shin et al. [28] which asked, “Where do you receive e-cigarette information from?”.

The next group of questions measured GPs’ confidence levels discussing e-cigarettes and their ability to assist patients with smoking cessation treatments on a five-point Likert scale revised from Nickels et al. [20] with selections varying from “not at all confident” to “very confident”.

The final set of questions focused on GPs' recommendations and prescribing intentions of e-cigarettes as a smoking cessation aid. One question was taken from Brett et al. [25] which asked, “Would your advice to patients about using e-cigarettes, or vaping, include any of the following?”. The rest of the questions in this subset assessed understanding of the new e-cigarette regulations in Australia that took effect from October 1, 2021 and included, “Have you ever recommended e-cigarettes to your patients for smoking cessation prior to the laws changing in October 1, 2021?” and “Since the recent changes to e-cigarette laws (October 1, 2021), have you recommended e-cigarettes to your patients for smoking cessation?”. The baseline survey (S1) and the follow-up survey (S2) can be found in the supplementary file.

### Statistical analysis

Qualtrics was used to create the online survey and data analyses were performed using SPSS Version 29 (IBM SPSS Statistics for Windows, Armonk, NY, 2021) and STATA 16 (STATA Corp, 2022). Socio-demographic characteristics were analysed in SPSS using Chi-Squared tests for categorical variables and independent samples T-tests or One-Way Analysis of Variance (ANOVA) for continuous variables. P-values less than 0.05 were considered to be statistically significant. We ran two sets of logistic regression analyses using STATA, the first set was a full sample of GPs during baseline study (T1), and the second set was for the follow-up (T2) group to address the study objectives to assess changes across a time of regulatory change in Australia. At T1, knowledge (total knowledge score), attitude (efficacy), behavioural (confidence), and social norm (colleagues) were regressed on GPs' recommendation of e-cigarettes to their patients after the recent changes to e-cigarette laws (October 1, 2021) using ordinal logistic regression analysis controlling for GPs years of practice/experience and country of education. During the T2 analysis, all the variables used in baseline analysis were regressed for the change in the intention of GPs recommendation of e-cigarettes to their patients in the past 12 months. In both sets of analysis, the age and sex of the GPs were adjusted. Covariates were studied for univariate and multivariate analysis, and the odds ratio (OR) and the adjusted OR (aOR) were reported with a 95% confidence interval (CI) and p values. Missing data of all variables was less than 5%.

## Results

Socio-demographic characteristics of participants is presented in Table 1. A total of *n* = 264 participants completed the survey at baseline (T1) and *n* = 94 at follow up (T2). At both T1 and T2 there were a larger number of female respondents (T1, *n* = 170, 64.4% vs. T2, *n* = 57, 60.6%). There were few participants aged less than thirty, and most respondents had fewer than 10 years of practice experience and worked in a group practice (T1, 70.1% vs. T2, 73.4%).

**Table 1:** Socio-demographic characteristics of respondents

	T1	Total *N* = 264 (%)	T2	Total *N* = 94 (%)
Gender	Male	94 (35.6)	Male	37 (39.4)
	Female	170 (64.4)	Female	57 (60.6)
Age*	20–29	15 (7.0)	20–29	4 (4.3)
	30–39	80 (37.2)	30–39	28 (29.8)
	40–49	59 (27.4)	40–49	28 (29.8)
	50–59	38 (17.7)	50–59	19 (20.2)
	60+	23(10.7)	60+	15 (16.0)
State^	VIC	60 (24.4)	**Has your postcode changed in the past 12 months?**	
	NSW	89 (36.2)	Yes	15 (16.0)
	SA	19 (7.7)	No	79 (84.0)
	WA	20 (8.1)		
	QLD	48 (19.5)		
	TAS	6 (2.4)		
	NT	4 (1.6)		
Years of practice^#+^	0–10	158 (60.3)	0–10	42 (46.2)
	11–20	54 (20.6)	11–20	21 (23.1)
	21–30	27 (10.3)	21–30	14 (15.4)
	30+	23 (8.8)	30+	14 (15.4)
Medical degree obtained	Australia	209 (79.2)	Australia	79 (84.0)
	International	55 (20.8)	International	15 (16.0)
Primary practice	Solo	9 (3.4)	Solo	4 (4.3)
	Group	185 (70.1)	Group	69 (73.4)
	Corporate	36 (13.6)	Corporate	11 (11.7)
	Aboriginal health services	16 (6.1)	Aboriginal health services	4 (4.3)
	Other	18 (6.8)	Other	6 (6.4)
Remoteness^	Metro	196 (79.7)		
	Inner regional	34 (13.8)		
	Outer regional	11 (4.5)		
	Remote or very remote	5 (2.0)		
Smoking status	Daily	3 (1.1)	Daily	0 (0.0)
	Less than weekly	3 (1.1)	Less than weekly	1 (1.1)
	Former smoker	30 (11.4)	Former smoker	16 (17.0)
	Never smoker	228 (86.4)	Never been a smoker	77 (81.9)
E-cigarette use**	Daily	5 (1.9)	Daily	3 (3.2)
	Less than weekly	7 (2.7)	Less than weekly	2 (2.2)
	Former e-cigarette user	4 (1.5)	Former e-cigarette user	3 (3.2)
	Never e-cigarette user	248 (93.9)	Never e-cigarette user	85 (91.4)

*n = 49 (18.6%) of participants did not complete this question

^*n* = 18 (6.8%) of participants did not complete this question

#*n* = 2 (0.8%) of participants did not complete this question

**n = 1 (1.1%) of participants did not complete this question (T2)

+*n* = 3 (3.2%) of participants did not complete this question (T2)

At T1 and T2 few GPs had recommended e-cigarettes to their patients for smoking cessation (T1 *n* = 34, 14.1%, T2 *n* = 20, 22.7%). Male GPs were more likely to have recommend e-cigarettes at both T1 and T2 (Table 2).

**Table 2:** Prescribing intentions by socio-demographics at T1 and T2

	T1				T2			
Since the recent changes have you recommended e-cigarettes to your patients?	Yes *N* = 34 (14.1%)	No *N* = 207 (85.9%)	Test Statistic	*p*-value	Yes *N* = 20 (22.7%)	No *N* = 68 (77.3%)	Test Statistic	*p*-value
Gender*^#^								
Male Female	*n* = 23 (25.8%) *n* = 11 (7.2%)	*n* = 66 (74.2%) *n* = 141 (92.8%)	χ^2^ = 16.036 df = 1	*p* = < 0.001	*n* = 14 (41.2%) *n* = 6 (11.1%)	*n* = 20 (58.8%) *n* = 48 (88.9%)	χ^2^ = 10.379 df = 1	*p* = 0.001
**Age^** ^**#**^								
20–29 30–39 40–49 50–59 60+	*n* = 5 (33.3%) *n* = 11 (13.9%) *n* = 7 (13.5%) *n* = 2 (6.1%) *n* = 4 (18.2%)	*n* = 10 (66.7%) *n* = 68 (86.1%) *n* = 45 (86.5%) *n* = 31 (93.9%) *n* = 18 (81.8%)	χ^2^ = 6.520 df = 4	*p* = 0.164	*n* = 0 (0.0%) *n* = 5 (19.2%) *n* = 8 (30.8%) *n* = 3 (17.6%) *n* = 4 (26.7%)	*n* = 4 (3.1%) *n* = 21 (80.8%) *n* = 18 (69.2%) *n* = 14 (82.4%) *n* = 11 (73.3%)	χ^2^ = 2.697 df = 4	*p* = 0.610
**Years of Practice** ^**+−**^								
0–10 11–20 21–30 30+	*n* = 22 (14.8%) *n* = 8 (16.0%) *n* = 0 (0.0%) *n* = 4 (19.0%)	*n* = 127 (85.2%) *n* = 42 (84.0%) *n* = 20 (100.0%) *n* = 17 (81.0%)	χ^2^ = 3.894 df = 3	*p* = 0.273	*n* = 9 (23.7%) *n* = 6 (28.6%) *n* = 0 (0.0%) *n* = 4 (28.6%)	*n* = 29 (76.3%) *n* = 15 (71.4%) *n* = 13(100.0%) *n* = 4 (71.4%)	χ^2^ = 4.596 df = 3	*p* = 0.204
Training* ^**#**^								
Australia International	*n* = 28 (14.8%) *n* = 6 (11.5%)	*n* = 161 (85.2%) *n* = 46 (88.5%)	χ^2^ = 0.361 df = 1	*p* = 0.548	*n* = 16 (21.3%) *n* = 4 (30.8%)	*n* = 59 (78.7%) *n* = 9 (69.2%)	χ^2^ = 0.562 df = 1	*p* = 0.454

*n = 23 (8.7%) of participants did not answer this question

#*n* = 6 (6.4%) of participants did not answer this question (T2)

^*n* = 63 (23.9%) of participants did not answer this question

+*n* = 24 (9.1%) of participants did not answer this question

-*n* = 8 (8.5%) of participants did not answer this question (T2)

### GPs’ knowledge, attitudes and social norms about e-cigarettes for smoking cessation

Australian GPs had limited levels of knowledge about e-cigarettes at T1 (x̄=2.7/6) and at T2 (x̄=3.4/6) (Table 3). GPs at both time points received their e-cigarette information primarily from scientific literature and the RACGP. Male GPs and those with an Australian medical qualification were the most knowledgeable about e-cigarettes at T1. At T2 the international medical graduates had similar levels of knowledge. GPs at T1 and T2 with the highest knowledge scores were the most confident when talking to patients about e-cigarettes (Table 3). At T2, GPs with the highest knowledge scores were also more likely to recommend e-cigarettes to patients, and those registered as authorised prescribers (Table 3).

**Table 3:** Knowledge about e-cigarettes by socio-demographics and prescribing intentions at T1 and T2

	T1 - *N*	T1 Mean (SD)	Statistic, *p*-value	T2- *N*	T2 Mean (SD)	Statistic, *p*-value
**Total average knowledge score**	*n* = 252	2.7 (SD = 1.5)		*n* = 91	3.4 (SD = 1.5)	
**Gender**						
Male Female	*n* = 88 *n* = 164	3.1 (SD = 1.6) 2.5 (SD = 1.3)	T(150) = 2.8, *p* = 0.005	*n* = 35 *n* = 56	3.8 (SD = 1.6) 3.1 (SD = 1.4)	T(64) = 2.1, *p* = 0.039
**Years of practice**						
0–10 11–20 21–30 30+	*n* = 152 *n* = 51 *n* = 27 *n* = 20	2.7 (1.5) 3.0 (1.4) 2.7 (1.7) 2.9 (1.4)	F = 0.6, *p* = 0.601	*n* = 39 *n* = 21 *n* = 14 *n* = 14	3.4 (1.6) 4.0 (1.3)* 2.4 (1.3)* 3.6 (1.2)	F = 3.5, *p* = 0.019
**Medical degree obtained**						
Australian graduate International graduate	*n* = 200 *n* = 52	2.9 (1.5) 2.3 (1.3)	T(250) = 2.5, *p* = 0.006	*n* = 77 *n* = 14	3.4 (1.4) 3.2 (2.0)	T(15.4) = 0.3, *p* = 0.741
**Confidence talking to patients about e-cigarettes^**						
*Not at all/somewhat not* *Neither/nor* *Very/somewhat*	*n* = 124 *n* = 54 *n* = 61	2.4 (1.3) 2.8 (1.5)* 3.3 (1.4)*	F = 7.7, p = < 0.001	*n* = 32 *n* = 14 *n* = 42	2.9 (1.4)* 3.1 (1.4) 3.8 (1.5)*	F = 3.6, *p* = 0.051
**I WOULD recommend using e-cigarettes or vaping^**						
Yes No	*n* = 149 *n* = 103	2.8 (1.5) 2.6 (1.3)	T(250) = 1.350, *p* = 0.359	*n* = 61 *n* = 30	3.6 (1.5) 2.8 (1.3)	T(89) = 2.472, *p* = 0.038
**Have you recommended e-cigarettes to your patients? ^**						
Yes No	*n* = 36 *n* = 203	3.1 (1.4) 2.7 (1.4)	T(237) = 1.8, *p* = 0.290	*n* = 19 *n* = 68	4.5 (1.3) 3.0 (1.4)	T(85) = 4.0, p = < 0.001
**Prescriber Pathway^**						
Registered prescriber Unsure if will register/prescribe Won’t prescribe	*n* = 44 *n* = 98 *n* = 97	3.0 (1.5) 2.5 (1.4) 2.9 (1.4)	F = 2.6, *p* = 0.752	*n* = 18 *n* = 30 *n* = 38	4.4 (1.1) 3.4 (1.5) 2.9 (1.5)	F = 6.4, *p* = 0.005

* Difference between groups on Post Hoc test; ^ Adjusted for gender and international qualification

There was a high degree of variability in the attitudes toward e-cigarettes for smoking cessation between the GPs in this study, and this variability strengthened between time points (Table 4). GPs who intended to prescribe e-cigarettes for smoking cessation held more positive attitudes toward them than those who would not prescribe them. Beliefs about e-cigarettes being addictive appeared to strengthen between T1 and T2, as did perceptions of the risk of harm for people who use e-cigarettes. Beliefs about e-cigarettes being more effective than other smoking cessation aids did not change between T1 and T2 (Table 4).

**Table 4:** Beliefs and attitudes towards e-cigarettes and prescribing intentions of e-cigarettes at T1 and T2

	T1				T2			
Since the recent changes have you recommended e-cigarettes to your patients?	Yes	No	Test Statistic	*p*-value	Yes	No	Test Statistic	*p*-value
E-cigs are a gateway to smoking*^								
*Strongly/somewhat disagree* *Neither/nor* *Strongly/somewhat agree*	*n* = 7 (21.2%) *n* = 16 (48.5%) *n* = 10 (30.3%)	*n* = 22 (10.7%) *n* = 68 (33.2%) *n* = 115 (56.1%)	χ^2^ = 8.1 df = 2	*p* = 0.018	*n* = 4 (20.0%) *n* = 5 (25.0%) *n* = 11 (55.0%)	*n* = 3 (4.4%) *n* = 10 (14.7%) *n* = 55 (80.9%)	χ^2^ = 7.1 df = 2	*p* = 0.029
E-cigs can be addictive*^								
*Strongly/somewhat disagree* *Neither/nor* *Strongly/somewhat agree*	*n* = 1 (3.0%) *n* = 4 (12.1%) *n* = 28 (84.8%)	*n* = 2 (1.0%) *n* = 16 (7.8%) *n* = 187 (91.2%)	χ^2^ = 1.7 df = 2	*p* = 0.425	*n* = 0 (0.0%) *n* = 0 (0.0%) *n* = 20 (100%)	*n* = 0 (0.0%) *n* = 0 (0.0%) *n* = 68 (100%)		
E-cigs can be regarded as a type of smoking cessation aid*^								
*Strongly/somewhat disagree* *Neither/nor* *Strongly/somewhat agree*	*n* = 1 (3.0%) *n* = 3 (9.1%) *n* = 29 (87.9%)	*n* = 63 (30.7%) *n* = 53 (25.9%) *n* = 89 (43.4%)	χ^2^ = 22.8 df = 2	p = < 0.001	*n* = 0 (0.0%) *n* = 0 (0.0%) *n* = 20 (100%)	*n* = 28 (41.2%) *n* = 17 (25.0%) *n* = 23 (33.8%)	χ^2^ = 27.1 df = 2	p = < 0.001
E-cigs can decrease the number of cigarettes smoked*^								
*Strongly/somewhat disagree* *Neither/nor* *Strongly/somewhat agree*	*n* = 0 (0.0%) *n* = 3 (9.1%) *n* = 30 (90.9%)	*n* = 25 (12.2%) *n* = 47 (22.9%) *n* = 133 (64.9%)	χ^2^ = 9.4 df = 2	*p* = 0.009	*n* = 0 (0.0%) *n* = 1 (5.0%) *n* = 19 (95.0%)	*n* = 11 (16.2%) *n* = 15 (22.1%) *n* = 42 (61.8%)	χ^2^ = 8.2 df = 2	*p* = 0.017
E-cigs can lower the risk of tobacco-related diseases*^								
*Strongly/somewhat disagree* *Neither/nor* *Strongly/somewhat agree*	*n* = 0 (0.0%) *n* = 4 (12.1%) *n* = 29 (87.9%)	*n* = 39 (19.0%) *n* = 77 (37.6%) *n* = 89 (43.4%)	χ^2^ = 23.0 df = 2	p = < 0.001	*n* = 0 (0.0%) *n* = 1 (5.0%) *n* = 19 (95.0%)	*n* = 21 (30.9%) *n* = 17 (25.0%) *n* = 30 (44.1%)	χ^2^ = 16.4 df = 2	p = < 0.001
E-cigs can help patients quit smoking*^								
*Strongly/somewhat disagree* *Neither/nor* *Strongly/somewhat agree*	*n* = 1 (3.0%) *n* = 4 (12.1%) *n* = 28 (84.8%)	*n* = 60 (29.3%) *n* = 65 (31.7%) *n* = 80 (39.0%)	χ^2^ = 24.5 df = 2	p = < 0.001	*n* = 0 (0.0%) *n* = 1 (5.0%) *n* = 19 (95.0%)	*n* = 27 (39.7%) *n* = 16 (23.5%) *n* = 25 (36.8%)	χ^2^ = 21.2 df = 2	p = < 0.001
E-cigs are safer than regular cigarettes*^								
*Strongly/somewhat disagree* *Neither/nor* *Strongly/somewhat agree*	*n* = 1 (3.0%) *n* = 7 (21.2%) *n* = 25 (75.8%)	*n* = 69 (33.7%) *n* = 64 (31.2%) *n* = 72 (35.1%)	χ^2^ = 21.5 df = 2	p = < 0.001	*n* = 0 (0.0%) *n* = 3 (15.0%) *n* = 17 (85.0%)	*n* = 24 (35.3%) *n* = 23 (33.8%) *n* = 21 (30.9%)	χ^2^ = 19.4 df = 2	p = < 0.001
E-cigs have adverse health effects* ^**#**^								
*Strongly/somewhat disagree* *Neither/nor* *Strongly/somewhat agree*	*n* = 1 (3.0%) *n* = 7 (21.2%) *n* = 25 (75.8%)	*n* = 9 (4.4%) *n* = 19 (9.3%) *n* = 177 (86.3%)	χ^2^ = 4.2 df = 2	*p* = 0.122	*n* = 0 (0.0%) *n* = 3 (15.8%) *n* = 16 (84.2%)	*n* = 1 (1.5%) *n* = 2 (2.9%) *n* = 65 (95.6%)	χ^2^ = 4.8 df = 2	*p* = 0.093
E-cigs are less harmful than regular cigarettes*^								
*Strongly/somewhat disagree* *Neither/nor* *Strongly/somewhat agree*	*n* = 0 (0.0%) *n* = 5 (15.2%) *n* = 28 (84.4%)	*n* = 47 (22.9%) *n* = 72 (35.1%) *n* = 86 (42.0%)	χ^2^ = 22.0 df = 2	p = < 0.001	*n* = 0 (0.0%) *n* = 2 (10.0%) *n* = 18 (90.0%)	*n* = 22 (32.4%) *n* = 20 (29.4%) *n* = 26 (38.2%)	χ^2^ = 17.1 df = 2	p = < 0.001
E-cig use is harmful for the health of the user*^								
*Strongly/somewhat disagree* *Neither/nor* *Strongly/somewhat agree*	*n* = 2 (6.1%) *n* = 8 (24.2%) *n* = 23 (69.7%)	*n* = 6 (2.9%) *n* = 26 (12.7%) *n* = 173 (84.4%)	χ^2^ = 4.2 df = 2	*p* = 0.120	*n* = 0 (0.0%) *n* = 3 (15.0%) *n* = 17 (85.0%)	*n* = 0 (0.0%) *n* = 2 (2.9%) *n* = 66 (97.1%)	χ^2^ = 4.2 df = 1	*p* = 0.041
E-cigs are carcinogenic*^								
*Strongly/somewhat disagree* *Neither/nor* *Strongly/somewhat agree*	*n* = 11 (33.3%) *n* = 17 (51.5%) *n* = 5 (15.2%)	*n* = 12 (5.9%) *n* = 122 (59.5%) *n* = 71 (34.6%)	χ^2^ = 25.9 df = 2	p = < 0.001	*n* = 8 (40.0%) *n* = 10 (50.0%) *n* = 2 (10.0%)	*n* = 4 (5.9%) *n* = 36 (52.9%) *n* = 28 (41.2%)	χ^2^ = 17.6 df = 2	p = < 0.001
E-cigs are more effective than other smoking cessation aids*^								
*Strongly/somewhat disagree* *Neither/nor* *Strongly/somewhat agree*	*n* = 10 (30.3%) *n* = 15 (45.5%) *n* = 8 (24.2%)	*n* = 127 (62.0%) *n* = 70 (34.1%) *n* = 8 (3.9%)	χ^2^ = 23.5 df = 2	p = < 0.001	*n* = 5 (25.0%) *n* = 11 (55.0%) *n* = 4 (20.0%)	*n* = 51 (75.0%) *n* = 15 (22.1%) *n* = 2 (2.9%)	χ^2^ = 18.3 df = 2	p = < 0.001

*n = 26 (9.8%) of participants did not answer these questions

^*n* = 6 (6.4%) of participants did not answer this question (T2)

#*n* = 7 (7.4%) of participants did not answer this question (T2)

GPs who perceived greater behavioural control, that is they had greater confidence in their abilities to talk with patients, answer patient questions and prescribe nicotine e-liquids, were more likely to have recommended e-cigarettes for smoking cessation at both T1 and T2 (Table 5).

**Table 5:** Behavioural control and prescribing intentions of e-cigarettes at T1 and T2

	T1				T2			
Since the recent changes have you recommended e-cigarettes to your patients?	Yes	No	Test Statistic	*p*-value	Yes	No	Test Statistic	*p*-value
**Confidence with** **Level of knowledge about e-cigarettes** ^**#+**^								
*Not at all/somewhat not* *Neither/nor* *Very/somewhat*	*n* = 6 (17.6%) *n* = 6 (17.6%) *n* = 22 (64.7%)	*n* = 112 (54.1%) *n* = 54 (26.1%) *n* = 41 (19.8%)	χ^2^ = 31.285 df = 2	p = < 0.001	*n* = 2 (10.0%) *n* = 1 (5.0%) *n* = 17 (85.0%)	*n* = 25 (36.8%) *n* = 15 (22.1%) *n* = 28 (41.2%)	χ^2^ = 11.886 df = 2	*p* = 0.003
**Ability to answer questions from patients about e-cigarettes** ^**^+**^								
*Not at all/somewhat not* *Neither/nor* *Very/somewhat*	*n* = 4 (11.8%) *n* = 9 (26.5%) *n* = 21 (61.8%)	*n* = 114 (55.3%) *n* = 48 (23.3%) *n* = 44 (21.4%)	χ^2^ = 28.986 df = 2	p = < 0.001	*n* = 2 (10.0%) *n* = 2 (10.0%) *n* = 16 (80.0%)	*n* = 27 (39.7%) *n* = 13 (19.1%) *n* = 28 (41.2%)	χ^2^ = 9.551 df = 2	*p* = 0.008
Ability to talk to patients about e-cigarettes* ^**+**^								
*Not at all/somewhat not* *Neither/nor* *Very/somewhat*	*n* = 6 (17.6%) *n* = 4 (11.8%) *n* = 24 (70.6%)	*n* = 117 (57.1%) *n* = 50 (24.4%) *n* = 38 (18.5%)	χ^2^ = 41.324 df = 2	p = < 0.001	*n* = 3 (15.0%) *n* = 0 (0.0%) *n* = 17 (85.0%)	*n* = 29 (42.6%) *n* = 13 (19.1%) *n* = 26 (38.2%)	χ^2^ = 13.989 df = 2	p = < 0.001
**Ability to prescribe nicotine e-liquid** ^**^+**^								
*Not at all/somewhat not* *Neither/nor* *Very/somewhat*	*n* = 11 (32.4%) *n* = 6 (17.6%) *n* = 17 (50.0%)	*n* = 168 (81.6%) *n* = 26 (12.6%) *n* = 12 (5.8%)	χ^2^ = 57.154 df = 2	p = < 0.001	*n* = 6 (30.0%) *n* = 3 (15.0%) *n* = 11 (55.0%)	*n* = 51 (75.0%) *n* = 11 (16.2%) *n* = 6 (8.8%)	χ^2^ = 21.903 df = 2	p = < 0.001

*n = 25 (9.5%) of participants did not answer this question

#*n* = 23 (8.7%) of participants did not answer this question

^*n* = 24 (9.1%) of participants did not answer this question

+*n* = 6 (6.4%) of participants did not answer this question (T2)

### GP intentions to prescribe nicotine containing e-cigarettes for smoking cessation

At T1 and T2, GPs’ intentions to prescribe nicotine containing e-cigarettes were not influenced by other GPs in their practice who were willing to prescribe nicotine e-liquids to their patients (Table 6). However, GPs at T1 were more likely to be influenced by practice ownership, colleagues and online GP groups to counsel, recommend and prescribe e-cigarettes to patients for smoking cessation compared to T2. In both T1 and T2, GPs were shown to be influenced by their patients and their own family members or friends to counsel, recommend and prescribe e-cigarettes for smoking cessation (Table 6).

**Table 6:** Social norms and prescribing intentions of e-cigarettes at T1 and T2

	T1				T2			
	Have you ever recommended e-cigs to your patients for smoking cessation prior to the laws changing in October 1, 2021?				In the past 12 months have you recommended e-cigs to your patients for smoking cessation?			
	Yes	No	Test Statistic	*p*-value	Yes	No	Test Statistic	*P*-value
**Are there other GPs in your practice that are willing to prescribe nicotine e-liquids to their patients?** ^**#**^ **-**								
Yes No Unsure	*n* = 6 (16.7%) *n* = 7 (19.4%) *n* = 23 (63.9%)	*n* = 21 (10.3%) *n* = 52 (25.6%) *n* = 130 (64.0%)	χ^2^ = 1.6 df = 2	*p* = 0.449	*n* = 6 (30.0%) *n* = 6 (30.0%) *n* = 8 (40.0%)	*n* = 6 (9.0%) *n* = 24 (35.8%) *n* = 37 (55.2%)	χ^2^ = 5.8 df = 2	*p* = 0.053
**How likely are the following groups to influence your decision to counsel**,** recommend and prescribe e-cigarettes to patients for smoking cessation?**								
Practice ownership* ^**+**^								
Unlikely Neutral Likely	*n* = 14 (37.8%) *n* = 12 (32.4%) *n* = 11 (29.7%)	*n* = 115 (56.7%) *n* = 60 (29.6%) *n* = 28 (13.8%)	χ^2^ = 7.0 df = 2	*p* = 0.030	*n* = 10 (50.0%) *n* = 6 (30.0%) *n* = 4 (20.0%)	*n* = 47 (69.1%) *n* = 15 (22.1%) *n* = 6 (8.8%)	χ^2^ = 3.0 df = 2	*p* = 0.233
**Senior Colleagues/GP supervisor** ^**#+**^								
Unlikely Neutral Likely	*n* = 6 (16.2%) *n* = 10 (27.0%) *n* = 21 (56.8%)	*n* = 67 (33.2%) *n* = 35 (17.3%) *n* = 100 (49.5%)	χ^2^ = 4.8 df = 2	*p* = 0.095	*n* = 5 (25.0%) *n* = 4 (20.0%) *n* = 11 (55.0%)	*n* = 30 (44.1%) *n* = 6 (8.8%) *n* = 32 (47.1%)	χ^2^ = 3.3 df = 2	*p* = 0.205
**Colleagues** ^**#+**^								
Unlikely Neutral Likely	*n* = 2 (5.6%) *n* = 11 (30.6%) *n* = 23 (63.9%)	*n* = 57 (28.1%) *n* = 33 (16.3%) *n* = 113 (55.7%)	χ^2^ = 10.0 df = 2	*p* = 0.006	*n* = 4 (20.0%) *n* = 4 (20.0%) *n* = 12 (60.0%)	*n* = 25 (36.8%) *n* = 7 (10.3%) *n* = 36 (52.9%)	χ^2^ = 2.6 df = 2	*p* = 0.258
**Online GP Groups** ^**^+**^								
Unlikely Neutral Likely	*n* = 3 (8.1%) *n* = 11 (29.7%) *n* = 23 (62.2%)	*n* = 64 (31.4%) *n* = 40 (19.6%) *n* = 100 (49.0%)	χ^2^ = 8.7 df = 2	*p* = 0.013	*n* = 4 (20.0%) *n* = 5 (25.0%) *n* = 11 (55.0%)	*n* = 24 (35.3%) *n* = 9 (13.2%) *n* = 35 (51.5%)	χ^2^ = 2.5 df = 2	*p* = 0.290
**Patients** ^**#+**^								
Unlikely Neutral Likely	*n* = 6 (16.2%) *n* = 8 (21.6%) *n* = 23 (62.2%)	*n* = 107 (53.0%) *n* = 52 (25.7%) *n* = 43 (21.3%)	χ^2^ = 28.0 df = 2	*p* = 0.000	*n* = 2 (10.0%) *n* = 4 (20.0%) *n* = 17 (70.0%)	*n* = 39 (57.4%) *n* = 15 (22.1%) *n* = 14 (20.6%)	χ^2^ = 19.3 df = 2	p = < 0.001
**Family Members or Friends** ^**#+**^								
Unlikely Neutral Likely	*n* = 15 (41.7%) *n* = 15 (41.7%) *n* = 6 (16.7%)	*n* = 145 (71.4%) *n* = 42 (20.7%) *n* = 16 (7.9%)	χ^2^ = 12.3 df = 2	*p* = 0.003	*n* = 8 (40.0%) *n* = 7 (35.0%) *n* = 5 (25.0%)	*n* = 48 (70.6%) *n* = 13 (19.1%) *n* = 7 (10.3%)	χ^2^ = 6.4 df = 2	*p* = 0.040

*n = 24 (9.1%) of participants did not answer this question

#*n* = 25 (9.5%) of participants did not answer this question

^*n* = 23 (8.7%) of participants did not answer this question

+*n* = 6 (6.4%) of participants did not answer this question (T2)

-*n* = 7 (7.4%) of participants did not answer this question (T2)

Table 7 shows the results from the multivariate logistic regression models of predictors of GPs’ recommendation of e-cigarettes for patients to support smoking cessation at baseline. GPs were asked about any recommendations they had made since regulations regarding nicotine containing e-cigarettes changed, and their intentions to recommend e-cigarettes to eligible patients in the future who are unable to quit smoking with other methods. Among GPs who had recommended e-cigarettes for smoking cessation, GPs who agreed that e-cigarettes can help patients quit smoking and who felt very or somewhat comfortable talking to patients about e-cigarettes were most likely to have recommended e-cigarettes for smoking cessation.

**Table 7:** Regression estimates for the GPs interviewed at baseline (T1, *N* = 264)

Variables	Since the recent changes to e-cig laws (October 1, 2021), have you recommended e-cigarettes to your patients for smoking cessation? (*N* = 264)		Would you recommend electronic cigarettes to smokers who failed to quit with other methods? (*N* = 238)	
	Univariate OR 95% CI; *p* value	Multivariate aOR* 95% CI; *p* value	Univariate OR 95% CI; *p* value	Multivariate aOR* 95% CI; *p* value
**Years of practice**				
< 10 years (*n* = 158)	1.86(0.60, 5.68);0.276	1.02(0.15, 6.95);0.983	**2.22(1.08**,** 4.55);0.029**	3.79(0.61, 8.33);0.216
10–20 years (*n* = 54)	2.00(0.56, 7.10);0.2.84	1.95 (0.41, 9.22);0.399	2.12(0.90, 4.96);0.083	0.50(0.17, 1.47);0.210
**Reference**: >20years (*n* = 50)	----			
**Medical degree obtained**				
International (*n* = 55)	1.26(0.49, 3.22);0.625	0.52(0.16, 1.65);0.269	0.67(0.35, 1.25);0.216	0.65(0.31, 1.35);0.253
**Reference**: Australia (*n* = 209)	----			
**Knowledge** Total Knowledge Score High (*n* = 136) **Reference**: Low (*n* = 116)	1.84(0.85, 3.98);0.120	0.46(0.87, 1.14);0.094	**2.35(1.39**,** 3.96);0.001**	**2.77(1.51**,** 5.05);0.001**
**E-cigs can help patients quit smoking**				
strongly/somewhat disagree(*n* = 61)	0.27(0.03, 2.56);0.260	1.98(0.04, 5.15);0.564	**0.23 (0.10**,** 0.51);<0.001**	**0.26(0.10**,** 0.64);0.004**
strongly/somewhat agree(*n* = 111)	**5.65 (1.88**,** 16.90);0.002**	**6.68 (1.87**,** 23.81);0.003**	**3.33 (1.73**,**6.39);<0.001**	**3.74(1.82**,**7.71);<0.001**
**Reference**: neither/nor(*n* = 71)	----			
**Ability to talk to patients about e-cigs**				
Not at all/somewhat not confident (*n* = 124)	1.57(0.42, 5.81);0.497	0.87(0.20, 3.68);0.850	0.89(0.47, 1.0);0.744	0.72(0.35, 1.47);0.371
Very/somewhat confident (*n* = 63)	**7.69(2.46**,** 24.01);<0.001**	**7.00(1.82**,**26.87);0.005**	1.72(0.81, 3.66);0.156	1.42(0.60, 3.34);0.419
**Reference**: neither/nor (*n* = 54)	**----**			
**Colleagues influence on prescribing e-cigs**				
Unlikely (*n* = 60)	**0.20(0.05**,** 0.80);0.024**	0.36 (0.08,1.57);0.176	**0.24(0.10**,** 0.57);0.001**	**0.27(0.10**,** 0.72);0.008**
Likely (*n* = 137)	0.74(0.31, 1.76);0.502	1.06(0.39,2.90);0.898	1.56(0.77, 3.15);0.210	1.25 (0.56, 2.76);0.575
**Reference**: Neutral (*n* = 44)	----			

Note: OR = Odds Ratio; aOR = adjusted OR; 95% CI = 95% Confidence intervals; *model adjusted for the age and sex; TKS = Total Knowledge Score; TKS grouped as high if the score is above median average (> 3) and score less than or equal to 3 considered as having low TKS

At baseline, among GPs who intended to recommend e-cigarettes in the future to people who smoke that were unsuccessful to quit with other methods, GPs who were more knowledgeable and believed e-cigarettes can help patients quit smoking had greater odds of recommending e-cigarettes for smoking cessation. Table 8 shows these predictors at follow up 12 months later. Knowledge score predicted if GPs had made a recommendation of e-cigarettes to patients in the past 12 months, however, years of practice, knowledge, belief that e-cigarettes can help patients quit smoking, and ability to talk to patients about e-cigarettes were all independent predictors of intention to recommend e-cigarettes to patients for smoking cessation in the future (Table 8).

**Table 8:** Regression estimates for the GPs interviewed at follow-up (T2, *N* = 94)

Variables	In the past 12 months have you recommended e-cigs to your patients for smoking cessation? (T2, *N* = 88)		Would you recommend electronic cigarettes to smokers who failed to quit with other methods? (T2, *N* = 87)	
	Univariate OR 95% CI; *p* value	Multivariate aOR* 95% CI; *p* value	Univariate OR 95% CI; *p* value	Multivariate aOR* 95% CI; *p* value
**Years of practice**				
< 10 years (*n* = 42)	1.78 (0.48, 6.53);0.382	7.37 (0.56, 96.67);0.128	**4.19(1.31**,** 11.90);0.016**	**18.38(1.21**,** 91.09);0.019**
10–20 years (*n* = 21)	2.29 (0.55, 9.53);0.251	5.54 (0.69, 44.39); 0.107	**3.81(1.04**,** 13.98); 0.043**	**8.68(1.37**,** 40.15);0.018**
**Reference**:>20years (*n* = 28)	----	----	----	----
**Medical degree obtained**				
International (*n* = 15)	1.63(0.44, 6.04);0.457	0.92 (0.26, 3.78);0.915	1.33(0.40, 3.55);0.637	1.05(0.25, 3.10);0.928
**Reference**: Australia (*n* = 79)	----	----	----	----
**Knowledge** Total Knowledge Score High (*n* = 63) **Reference**: Low (*n* = 28)	**10.46(1.31**,**83.19);0.026**	**10.88(1.29**,**91.51);0.026**	**2.51(1.08**,** 5.80);0.032**	**2.41(1.03**,** 5.61);0.042**
**E-cigs can help patients quit smoking**				
strongly/somewhat disagree (*n* = 27)	----	----	0.18(0.01, 1.89);0.163	0.20(0.07, 2.01);0.185
strongly/somewhat agree (*n* = 46)	**12.15 (1.47**, 99.91**);0.020**	8.32 (0.92, 75.27);0.059	**11.12(2.39**,** 38.75);<0.001**	**13.55(2.48**,**53.18);0.001**
**Reference**: neither/nor (*n* = 17)	**----**	----	**----**	**----**
**Ability to talk to patients about e-cigs**				
Not at all/somewhat not confident (*n* = 32)	----	----	1.4(0.24, 7.92);0.704	0.99(0.21, 7.34); 0.993
Very/somewhat confident (*n* = 43)	----	----	**7.64 (1.48**,** 39.28); 0.015**	**8.59(1.97**,** 48.78);0.015**
**Reference**: neither/nor (*n* = 14)	----	----	----	----
**Colleagues influence on prescribing e-cigs**				
Unlikely (*n* = 30)	0.28(0.05, 1.41);0.123	0.30(0.53, 19.43);0.199	0.58(0.13, 2.60);0.408	0.72(0.13, 3.05);0.690
Likely (*n* = 49)	0.58(1.42, 2.34);0.448	0.56(0.12, 7.98); 0.464	1.75(0.45, 6.76);0.417	1.72(0.43, 6.81);0.435
**Reference**: Neutral (*n* = 11)	----	----	----	----

Note: OR = Odds Ratio; aOR = adjusted OR; 95% CI = 95% Confidence intervals; *model adjusted for the age and sex; TKS = Total Knowledge Score; TKS grouped as high if the score is above median average (> 3) and score less than or equal to 3 considered as having low TKS

## Discussion

To the best of our knowledge this is the first study in Australia to have explored Australian GPs’ knowledge, attitudes, beliefs and intentions to prescribe nicotine containing e-cigarettes for smoking cessation. In this group of participants, we found socio-demographic and training characteristics explained differences in prescribing intention and that GPs who had greater confidence in their ability to talk with patients, answer patient questions and prescribe e-cigarettes, where most likely to recommend e-cigarettes for smoking cessation. Australian GPs’ knowledge about e-cigarettes remained limited with the need of further education. They mainly obtain information about e-cigarettes from scientific literature and the RACGP. Prescribing intentions were predicted by attitudes but not by the presence of other GPs in their practice that were willing to prescribe e-cigarettes. GPs were influenced by patients and their own family members or friends to counsel, recommend and prescribe e-cigarettes for smoking cessation to their patients.

A small number of GPs in our study were willing to recommend e-cigarettes to their patients at both T1 (14.1%) and T2 (22.7%) with male GPs being more inclined to recommend them. Similar findings internationally were found among physicians in Poland (11.5%) and Thailand (13.3%) agreeing that e-cigarettes should be recommended as alternative smoking cessation aids [31, 33], and male physicians in the US were more motivated to recommend e-cigarettes with 30% of physicians stating they had recommended them to their patients [34]. In other studies, 30% of physicians in the US were keen to recommend e-cigarettes to assist smoking cessation and 37% stated they would recommend them to decrease the number of cigarettes smoked [20]. Furthermore, physicians in China, US and the UK report a willingness to recommend e-cigarettes to patients as a temporary measure and a partial replacement, or as a form of harm reduction, to patients who are unable to quit smoking through alternative smoking cessation methods and to patients with co-morbidities [25, 26, 35].

There has been an ongoing global debate about whether or not e-cigarettes are an effective method for smoking cessation with concerns about not enough research and empirical evidence to support e-cigarettes as a smoking cessation alternative. Recent evidence from a Cochrane review on e-cigarettes as a smoking cessation aid found that people who smoke were more likely to quit smoking at 6 months using nicotine containing e-cigarettes compared to NRTs, e-cigarettes without nicotine or having no behavioural support [4]. E-cigarettes can be utilised by clinicians to help adults stop smoking, especially those who are unable to quit with pharmaceutical evidence-based treatments, however some GPs are sceptical of the evidence base supporting e-cigarette use as a smoking cessation aid and are reluctant, to recommend e-cigarettes for smoking cessation [14, 22, 27, 29, 31, 35–36].

Whilst many studies from around the world reported insufficient knowledge among physicians and those who felt they had low self-efficacy to talk to patients or give advice to patients about e-cigarettes [22, 25–27, 31, 36–37], our findings showed that Australian GPs were poorly informed about e-cigarettes at T1 and scored only slightly higher at T2. GPs with a higher knowledge score felt more confident in their ability to talk to patients about e-cigarettes and GPs who had registered to be a prescriber of e-cigarettes had higher knowledge scores at both T1 and T2. This suggests that GPs who knew more about e-cigarettes and had greater awareness about e-cigarettes to support smoking cessation were more inclined to register to prescribe e-cigarettes to their patients. Our findings were in line with other studies in the US where physicians who reported having more knowledge about e-cigarettes were more likely to recommend them to patients for smoking cessation [17].

In our study, GPs with more positive attitudes towards the role of e-cigarettes in smoking cessation and who agreed that e-cigarettes can be a type of smoking cessation aid were more likely to prescribe e-cigarettes to their patients to quit smoking. These findings align with previous literature about physicians’ attitudes and beliefs that e-cigarettes can limit the number of cigarettes smoked [20, 26], can lower the risk of tobacco-related diseases [20, 26], can be regarded as a type of smoking cessation aid or harm reduction tool [26, 35, 38, 39], can help with quitting smoking [36, 38–40] and are safer and less harmful than smoking cigarettes [26, 27, 38, 39] are more likely to prescribe e-cigarettes to patients for smoking cessation.

GPs from our study who reported being confident in their ability to prescribe e-cigarettes to their patients, answer patient questions and talk to patients about e-cigarettes were more likely to prescribe e-cigarettes to their patients for smoking cessation. Our findings mirrored international studies and found that physicians who felt more comfortable and confident having discussions with their patients and answering their questions were willing to recommend e-cigarettes as an alternative smoking cessation aid [20, 25, 26, 35].

Finally, we looked at GPs’ perceptions of social norms among their peers to find out whether or not GPs were influenced to counsel, recommend and prescribe e-cigarettes to their patients by social groups around them. Our findings suggest that GPs who had recommended or prescribed ENDS to their patients for smoking cessation were not influenced by other GPs in their work place but, they tended to be influenced by patients, their family members or friends. Some of our findings are in line with a study conducted by Erku et al. [41], which found that beliefs about ENDS among health care providers were influenced by patients’ experiences, and through media stories. Our study did not assess whether GPs were influenced by the media in their prescribing or recommending of e-cigarettes, however this could be explored in future studies.

### Strengths and limitations

There are a number of limitations present in this study. Responses at T1 were unable to be linked to responses at T2. It should be acknowledged that the analysis presents a descriptive analysis at two time points and is not a longitudinal analysis. While our survey was available to GPs within Australia it was not a representative national sample, and GPs with an interest in e-cigarettes may have been more likely to participate. The survey population tended to be younger than the national GP average age (50.6 years) [42] which may impact the support for vaping as this tends to be greater amongst younger people in Australia. Furthermore, only a third of GPs completed the survey at the 12-month follow up, as many participants from T1 did not provide their contact details to be surveyed for T2, and some didn’t respond to reminder emails to complete the survey. Moreover, GPs completed the survey subjectively, responses were self-reported and there was a potential for self-selection bias. Not every GP completed the full survey with answers missing to some of the questions which may have affected the findings. Regulations in Australia continue to change and the prescription model for e-cigarettes in Australia may be amended in the future [10]. Thus, care should be taken in generalising these findings to the entire GP population in Australia and attitudes may have changed following the recent changes to the regulation of e-cigarettes for smoking cessation. In addition, there has been significant misinformation about e-cigarettes and the potential role in smoking cessation which may have influenced changes between T1 and T2 which were not measured in this study [43].

## Conclusion

This is the first survey in Australia to have assessed and explored Australian GPs' knowledge, attitudes, beliefs and prescribing intentions for e-cigarettes as smoking cessation aids. These findings reflect GPs' attitudes and intentions following the introduction of regulations impacting access to e-cigarettes and nicotine e-liquids from GPs and other healthcare physicians by prescription that came into effect in Australia from 1 October 2021. Our results show that GPs with a higher knowledge score on e-cigarettes, GPs that had registered to be a prescriber, and those with greater confidence and with positive attitudes on e-cigarettes were more likely to prescribe and recommend e-cigarettes to their patients as a form of smoking cessation. These findings have important implications for the success of the regulatory model that is being imposed in Australia. Further regulations strengthening the prescription model of e-cigarette access have been introduced in Australia impacting other professional groups such as pharmacists and further studies are needed to understand the impact these additional regulations may have had on GPs' prescribing intentions. GP education is vital if they are to be gatekeepers of this role and prescribing in line with international evidence-based best practice guidelines [23, 44]. This highlights that implementation of policy change requires development of strategies to educate and support GPs and other health professionals (e.g. pharmacists) to consult with patients about smoking cessation alongside regulatory change.

## Electronic supplementary material

Below is the link to the electronic supplementary material.

Supplementary Material 1

Supplementary Material 2

## Data Availability

No datasets were generated or analysed during the current study.

## Abbreviations

E-cigarettes: Electronic cigarettes
ENDS: Electronic nicotine delivery systems
GPs: General practitioners
NRTs: Nicotine replacement therapies
RACGP: Royal Australian College of General Practitioners
TGA: Therapeutics Good Australia
TPB: Theory of Planned Behaviour

## References

[1] World Health Organization. *Tobacco*. 31 July 2023 [cited 2024 22 September]; Available from: https://www.who.int/news-room/fact-sheets/detail/tobacco#:~:text=Key%20facts,%2D%20and%20middle%2Dincome%20countries

[2] World Health Organization. WHO report on the global tobacco epidemic, 2023: protect people from tobacco smoke. 2023 [cited 2024 22 September]; Available from: https://iris.who.int/bitstream/handle/10665/372043/9789240077164-eng.pdf?sequence=1

[3] Dai X, Gakidou E, Lopez AD. Evolution of the global smoking epidemic over the past half century: strengthening the evidence base for policy action. Tob Control. 2022;31(2):129–37. 35241576 10.1136/tobaccocontrol-2021-056535

[4] Lindson N, et al. Electronic cigarettes for smoking cessation. Cochrane Database Syst Rev. 2024;1(1):CD010216. 38189560 10.1002/14651858.CD010216.pub8 PMC10772980

[5] Jerzynski T, Stimson GV. Estimation of the global number of vapers: 82 million worldwide in 2021. Drugs Habits Social Policy. 2023;24(2):91–103.

[6] Jerzynski T, et al. Estimation of the global number of e-cigarette users in 2020. Harm Reduct J. 2021;18(1):109. 34688284 10.1186/s12954-021-00556-7 PMC8541798

[7] World Health Organization. *Electronic cigarettes (E-cigarettes)*. 5 May 2024 [cited 2024 26 December]; Available from: https://www.who.int/publications/i/item/WPR-2024-DHP-001

[8] Therapeutic Goods Administration. Nicotine vaping laws are changing. 3. September 2021 [cited 2024 20 March]; Available from: https://www.tga.gov.au/news/blog/nicotine-vaping-laws-are-changing

[9] Australian Institute of Health and Welfare. *National Drug Strategy Household Survey 2022–2023*. 29 February 2024 [cited 2024 22 September]; Available from: https://www.aihw.gov.au/reports/illicit-use-of-drugs/national-drug-strategy-household-survey/contents/tobacco-and-e-cigarettes-vapes

[10] Therapeutic Goods Administration. Vapes: information for prescribers. 1. October 2024 [cited 2024 12 November]; Available from: https://www.tga.gov.au/resources/resource/guidance/vapes-information-prescribers#:~:text=Vapes%2520are%2520unapproved%2520goods%26text=A%2520valid%2520prescription%2520is%2520required,be%2520supplied%2520in%2520pharmacy%2520settings

[11] Jongebloed H, et al. The role of general practice nurses in supporting people to quit smoking: A qualitative study. PLoS ONE. 2024;19(7):e0306555. 39024273 10.1371/journal.pone.0306555 PMC11257311

[12] Royal Australian College of General Practitioners. Supporting smoking and vaping cessation: A guide for health professionals. 2024 [cited 2024 12 November]; Available from: https://www.racgp.org.au/getmedia/924ba55d-dc47-41f9-bf5b-7a4cf9e19963/RACGP-NVP-and-Vaping-Cessation-September-2024.pdf.aspx

[13] Therapeutic Goods Administration. *New vaping laws to commence 1 July 2024*. 28 June 2024 [cited 2024 22 September]; Available from: https://www.tga.gov.au/news/media-releases/new-vaping-laws-commence-1-july-2024#:~:text=The%2520domestic%2520manufacture%252C%2520supply%2520and,to%2520distribute%2520and%2520supply%2520vapes

[14] Selamoglu M, et al. Why do we have to be the gatekeepers?’ Australian general practitioners’ knowledge, attitudes and prescribing intentions on e-cigarettes as a smoking cessation aid. BMC Prim Care. 2024;25(1):53. 38326738 10.1186/s12875-024-02292-w PMC10848430

[15] Morphett K, et al. Evaluating the implementation of a prescription only regulatory model for nicotine vaping products: A qualitative study on the experiences and views of healthcare professionals. Int J Drug Policy. 2024;125:104353. 38364356 10.1016/j.drugpo.2024.104353

[16] Selamoglu M, et al. General practitioners’ knowledge, attitudes, beliefs and practices surrounding the prescription of e-cigarettes for smoking cessation: a mixed-methods systematic review. BMC Public Health. 2022;22(1):2415. 36550439 10.1186/s12889-022-14696-3 PMC9784030

[17] Egnot E, Jordan K, Elliott JO. Associations with resident physicians’ early adoption of electronic cigarettes for smoking cessation. Postgrad Med J. 2017;93(1100):319–25. 27697895 10.1136/postgradmedj-2016-134058

[18] El-Shahawy O, Brown R, Elston Lafata J. Primary Care Physicians’ Beliefs and Practices Regarding E-Cigarette Use by Patients Who Smoke: A Qualitative Assessment. Int J Environ Res Public Health. 2016. 13(5). 10.3390/ijerph13050445 PMC4881070 27128928

[19] Mughal F, Rashid A, Jawad M. Tobacco and electronic cigarette products: awareness, cessation attitudes, and behaviours among general practitioners. Prim Health Care Res Dev. 2018;19(6):605–9. 29880076 10.1017/S1463423618000166 PMC6692824

[20] Nickels AS, et al. Beliefs, practices, and Self-efficacy of US physicians regarding smoking cessation and electronic cigarettes: A National survey. Nicotine Tob Res. 2017;19(2):197–207. 27613879 10.1093/ntr/ntw194

[21] Stepney M, Aveyard B, Begh R. GPs’ and nurses’ perceptions of electronic cigarettes in England: A qualitative interview study. Br J Gen Pract. 2019. 69(678). 10.3399/bjgp18X699821 PMC6301358 30397013

[22] Selamoglu M, et al. Perceptions of family physicians in Istanbul about e-cigarettes as smoking cessation aids: a qualitative study. Addict Sci Clin Pract. 2024;19(1):99. 39736756 10.1186/s13722-024-00532-z PMC11684242

[23] National Institute for Health and Care Excellence. *Tobacco: preventing uptake, promoting quitting and treating dependence*. 21 Februrary 2024 [cited 2024 26 December]; Available from: https://www.nice.org.uk/guidance/ng209 36745727

[24] Jackson SE, Garnett C, Brown J. Prevalence and correlates of receipt by smokers of general practitioner advice on smoking cessation in England: a cross-sectional survey of adults. Addiction. 2021;116(2):358–72. 32648976 10.1111/add.15187 PMC8432152

[25] Brett J, et al. Electronic cigarettes as a smoking cessation aid for patients with cancer: beliefs and behaviours of clinicians in the UK. BMJ Open. 2020;10(11):e037637. 33444179 10.1136/bmjopen-2020-037637 PMC7678366

[26] Feng Y et al. Beliefs, attitudes, and confidence to deliver electronic cigarette counseling among 1023 Chinese physicians in 2018. Int J Environ Res Public Health. 2019. 16(17). 10.3390/ijerph16173175 PMC6747414 31480401

[27] Moysidou A et al. Knowledge and perceptions about nicotine, nicotine replacement therapies and electronic cigarettes among healthcare professionals in Greece. Int J Environ Res Public Health. 2016. 13(5). 10.3390/ijerph13050514 PMC4881139 27213421

[28] Shin DW, et al. Lung cancer specialist physicians’ attitudes towards e-cigarettes: A nationwide survey. PLoS ONE. 2017;12(2):e0172568. 28235068 10.1371/journal.pone.0172568 PMC5325291

[29] Van Gucht D, Baeyens F. Health professionals in Flanders perceive the potential health risks of vaping as lower than those of smoking but do not recommend using e-cigarettes to their smoking patients. Harm Reduct J. 2016;13(1):22. 27342543 10.1186/s12954-016-0111-4 PMC4919883

[30] Walsberger S, Havill M. NSW Community Behaviours, Beliefs & Attitudes Towards E-Cigarettes: Results of an Online Survey. Cancer Council NSW. 2015 [cited 2022 March 15]; Available from: https://www.cancercouncil.com.au/wp-content/uploads/2020/05/150528_ecig_srv_results_report_FINAL_for-distribution.pdf

[31] Zgliczynski WS, et al. Knowledge and beliefs of E-Cigarettes among physicians in Poland. Med Sci Monit. 2019;25:6322–30. 31439826 10.12659/MSM.916920 PMC6719564

[32] Ajzen I. The theory of planned behavior. Organ Behav Hum Decis Process. 1991;50(2):179–211.

[33] Chinwong S et al. Electronic Cigarettes and Tobacco Product Cessation: A Survey of Healthcare Providers’ Opinions on Safety and Recommendation. Healthc (Basel). 2024. 12(14). 10.3390/healthcare12141410 PMC11275567 39057553

[34] Steinberg MB, Giovenco DP, Delnevo CD. Patient-physician communication regarding electronic cigarettes. Prev Med Rep. 2015;2:96–8. 26844056 10.1016/j.pmedr.2015.01.006 PMC4721470

[35] Salloum RG, et al. Primary care physician perspectives on recommending E-cigarettes to smokers: a Best-Worst discrete choice experiment. J Gen Intern Med. 2021;36(11):3353–60. 33523343 10.1007/s11606-021-06615-w PMC8606483

[36] Zhou SS, Baptist AP. Electronic cigarettes: how confident and effective are allergists, pulmonologists, and primary care physicians in their practice behavior? Allergy Asthma Proc. 2020;41(3):192–7. 32375963 10.2500/aap.2020.41.200009

[37] Koprivnikar H, Zupanic T, Farkas JL. Beliefs and practices regarding electronic cigarettes in smoking cessation among healthcare professionals in Slovenia. Tob Prev Cessat. 2020;6:3. 32548340 10.18332/tpc/115029 PMC7291891

[38] Sharifi H, et al. Knowledge, attitude and practice of e-cigarettes among healthcare professionals and smoking cessation volunteers. Minerva Pneumologica. 2019;58(2):64–9.

[39] Tanriover O, et al. Do family physicians perceive electronic cigarette use as a harm reduction strategy for smokers? A survey from Istanbul. Prim Health Care Res Dev. 2022;23:e15. 35307043 10.1017/S1463423622000056 PMC8991075

[40] Talley B, et al. Addiction, cessation, & harm reduction: primary care provider knowledge & perceptions of electronic nicotine delivery system. Osteopath Family Physician. 2017;9(2):10–6.

[41] Erku DA, et al. Beliefs and Self-reported practices of health care professionals regarding electronic nicotine delivery systems: A Mixed-Methods systematic review and synthesis. Nicotine Tob Res. 2020;22(5):619–29. 30938442 10.1093/ntr/ntz046

[42] Department of Health and Aged Care. *Summary Statistics, Medical Profession*. 22 April 2024 [cited 2024 26 December]; Available from: https://hwd.health.gov.au/resources/data/summary-mdcl.html

[43] Janmohamed K, et al. Interventions to mitigate vaping misinformation: A Meta-Analysis. J Health Communication. 2022;27(2):84–92. 35220901 10.1080/10810730.2022.2044941

[44] Ministry of Health NZ. Regulatory guidelines for retailers of vaping and other notifiable products. 20. December 2024 [cited 2024 26 December]; Available from: https://www.health.govt.nz/regulation-legislation/vaping-herbal-smoking-and-smokeless-tobacco/requirements/regulatory-guidelines#:~:text=All%2520vaping%2520products%2520sold%2520in%2520New%2520Zealand%2520must%2520contain%2520a%2520removable%2520battery.%26text=Child%252Dsafety%2520mechanisms-,All%2520vaping%2520products%2520sold%2520in%2520New,have%2520a%2520child%2520safety%2520mechanism.%26text=Flavours-,All%2520vaping%2520products%2520sold%2520in%2520New,comply%2520with%2520variant%2520name%2520restrictions.%26text=Vaping%2520substances%2520must%2520not%2520exceed,limit%2520of%252020%2520mg%252Fml

