# Supplementary material for: Australian general practitioners’ knowledge, attitudes and prescribing intentions for e-cigarettes as a smoking cessation aid: a nationwide baseline and 12-month follow up survey
Source: Harm Reduct J. 2025 Mar 18;22:35. doi: 10.1186/s12954-025-01175-2 (PMC11916968; doi:10.1186/s12954-025-01175-2)
Supplement: Supplementary file 2 — Supplementary Material 2 [file 12954_2025_1175_MOESM2_ESM.pdf]

12-month follow up survey on the perceptions of Australian GPs on e-cigarettes as smoking cessation aids

Q1. What is your age (years)?

____

Q2. What is your gender?

- ○ Male
- ○ Female
- ○ Non-binary / gender diverse
- ○ My gender identity isn't listed. I identify as:__________________
- ○ Prefer not to say

Q3. Has your postcode of the clinic where you work at changed in the past 12 months?

____

If Yes, what was the postcode of your primary place of practice 12 months ago?

____

Q4. Thinking of your primary place of practice, is this best described as

- ◯ Solo practice
- ◯ Group practice
- ◯ Corporate practice
- ◯ Aboriginal health services
- ◯ Other (please specify) ____

Q5. How many years have you worked as a GP?

____

Q6. In which country did you receive your medical training?

____

Q7. What qualifications do you hold (tick all that apply)?

- ☐ FRACGP
- ☐ ACRRM
- ☐ Current GP registrar/ PEP
- ☐ MD/PhD
- ☐ MPH
- ☐ Other (please specify) ____

Q8. In the past 12 months, have you smoked cigarettes, pipes or other tobacco products?

- ◯ Daily
- ◯ At least once a week
- ◯ Less than weekly
- ◯ Not at all but I have been a regular smoker in the past
- ◯ Not at all and I have never been a regular smoker

Q9. In the past 12 months have you vaped or used e-cigarettes?

- ◯ Daily
- ◯ At least once a week
- ◯ Less than weekly
- ◯ Not at all but I have been a regular e-cigarette user in the past
- ◯ Not at all and I have never been a regular e-cigarette user

Q10. In your opinion which of the following are correct concerning e-cigarettes?

	Yes	Unsure	No
They contain tobacco	○	○	✓
There is combustion	○	○	✓
E-liquid ingredients are approved for inhalation	○	○	✓
Working temperature is lower than in tobacco cigarettes	✓	○	○
They have official quality certificates	○	○	✓
There are e-cigarettes without nicotine	✓	○	○

Q11. The following questions ask about your knowledge, beliefs and the safety and efficacy of e-cigarettes in the context of smoking cessation. Please indicate your response for each question.

	Strongly disagree	Somewhat disagree	Neither agree nor disagree	Somewhat agree	Strongly agree
It is part of my responsibility as a GP to make sure that my patients get the help they need to quit smoking	○	○	○	○	○
GP advice to quit is effective in helping patients quit smoking	○	○	○	○	○
Medications such as varenicline (champix)/bupropion (zyban)/nicotine gum/patches are effective in helping patients quit smoking	○	○	○	○	○
E-cigarettes are a gateway to smoking	○	○	○	○	○
E-cigarettes can be addictive	○	○	○	○	○
E-cigarettes can be regarded as a type of smoking cessation aid	○	○	○	○	○
E-cigarettes can decrease the number of cigarettes smoked	○	○	○	○	○
E-cigarettes can lower the risk of tobacco-related diseases	○	○	○	○	○
E-cigarettes can help patients quit smoking	○	○	○	○	○

E-cigarettes are safer than regular cigarettes	○	○	○	○	○
E-cigarettes have adverse health effects	○	○	○	○	○
E-cigarettes are less harmful than regular cigarettes	○	○	○	○	○
E-cigarette use is harmful for the health of the user	○	○	○	○	○
E-cigarette aerosol is harmful for people in the vicinity of the users	○	○	○	○	○
E-cigarettes are carcinogenic	○	○	○	○	○
E-cigarettes are more effective than other smoking cessation treatments	○	○	○	○	○

Q12. Where do you receive e-cigarette information from? (Select all that apply)

- ☐ Scientific literature
- ☐ RACGP
- ☐ Government reports/websites
- ☐ Non-government organisations
- ☐ Media (TV, radio, newspaper, internet)
- ☐ Social media
- ☐ Patients
- ☐ Healthcare colleagues
- ☐ E-cigarette companies/retailers
- ☐ None of these/have not received information about e-cigarettes
- ☐ Other (please specify) ____

Q13. How likely are the following groups to influence your decision to counsel, recommend and prescribe e-cigarettes to patients for smoking cessation?

	Extremely unlikely	Somewhat unlikely	Neither likely nor unlikely	Somewhat likely	Extremely likely
Practice ownership	○	○	○	○	○
Senior colleagues/GP supervisor	○	○	○	○	○
Colleagues	○	○	○	○	○
Online GP groups	○	○	○	○	○
Patients	○	○	○	○	○
Family members or friends	○	○	○	○	○
Other (please specify)	○	○	○	○	○

Q14. Please indicate your level of confidence for each question.

	Not at all confident	Somewhat not confident	Neither confident nor not confident	Somewhat confident	Very confident
Your ability to talk with your patients about their smoking?	○	○	○	○	○
Your knowledge about medications used to help patients quit smoking?	○	○	○	○	○
Your ability to help your patients stop smoking?	○	○	○	○	○
Your level of knowledge about e-cigarettes	○	○	○	○	○
Your ability to answer questions from patients about e-cigarettes	○	○	○	○	○
Your ability to talk to your patients about e-cigarettes for smoking cessation	○	○	○	○	○
Your ability to prescribe nicotine e-liquid in line with current guidelines	○	○	○	○	○

Q15. Would your advice to patients about using e-cigarettes, or vaping, include any of the following (please tick all that apply)

- ☐ I would say that some patients find using e-cigarettes, or vaping, helpful to stop smoking tobacco
- ☐ I would only recommend using e-cigarettes, or vaping, as a secondary approach to stop smoking tobacco (i.e. if all other medicinal therapies have failed)
- ☐ I would let the patient choose whether or not they want to use e-cigarettes, or vape, to stop smoking tobacco
- ☐ I would recommend using e-cigarettes, or vaping, to my patients as a first line therapy for stopping smoking tobacco
- ☐ I would recommend using e-cigarettes, or vaping, to tobacco smokers who do not intend to quit or who have declined the offer of help
- ☐ Other advice (please specify) ____
- ☐ I would not offer any advice about using e-cigarettes, or vaping to patient
- ☐ I do not recommend using e-cigarettes, or vaping, to patients

Q16. In the past 12 months have you recommended e-cigarettes to your patients for smoking cessation?

- ○ Yes
- ○ No

Q17. If no, what are the reasons (select all that apply)

- ☐ No suitable patients
- ☐ Time
- ☐ Reimbursement too low
- ☐ Not registered to prescribe
- ☐ Other (please specify) ____

Q18. In the past 12 months, from the options below, have you registered to any of the pathways to prescribe nicotine e-liquid to your patients for smoking cessation?

- ○ Streamline Authorised Prescriber
- ○ Special Access Scheme B
- ○ Personal Importation Scheme
- ○ I have not registered yet, as I am unsure on which pathway I will use
- ○ I have not registered yet, as I am unsure whether I will or won't prescribe e-cigarettes
- ○ I have not registered yet, as I have not had any suitable patients
- ○ I won't be prescribing e-cigarettes

Q19. Would you recommend e-cigarettes as a substitute to smokers who would refuse to take medications for smoking cessation?

- ◯ Yes
- ◯ No
- ◯ Unsure

Q20. Would you recommend electronic cigarettes to smokers who failed to quit with other methods?

- ◯ Yes
- ◯ No
- ◯ Unsure

Q21. Are there other GPs in your practice that are willing to prescribe nicotine e-liquid to their patients?

- ◯ Yes
- ◯ No
- ◯ Unsure

If there is anything further you would like to share about your experiences or concerns discussing e-cigarettes with your patients please use the text box below.

____

____

____

____

____
